# A Novel Method for the Preparation of Poly (Acrylamide-co-Acrylonitrile) Upper Critical Solution Temperature Thermosensitive Hydrogel by the Partial Dehydration of Acrylamide Grafted Polypropylene Sheets

**DOI:** 10.3390/gels8060345

**Published:** 2022-05-31

**Authors:** Yi Ling, Liuyuchen Chen, Mingjun Huang, Cheng Zhou, Liming Yang, Hejingying Niu, Li Su, Yuejiao Yang, Rogério P. Pirraco, Rui L. Reis, Jie Chen

**Affiliations:** 1 Department of Chemical Engineering and Technology, School of Environmental and Chemical Engineering, Shanghai University, Shangda Road 99, Shanghai 200444, China; tangyuf@siom.ac.cn (Y.L.); ychen761@uottawa.ca (L.C.); mingjun.huang@wdpharma.com (M.H.); zhoucheng@sqi.org.cn (C.Z.); yanglm@shu.edu.cn (L.Y.); niuhjy@shu.edu.cn (H.N.); yuejiao.yang@unitn.it (Y.Y.); 2 School of Life Sciences, Shanghai University, Shangda Road 99, Shanghai 200444, China; suli1020@shu.edu.cn; 3 BIOtech Research Center, Department of Industrial Engineering, University of Trento, Via Delle Regole 101, 38123 Trento, Italy; 4 3B’s Research Group—Biomaterials, Biodegradables and Biomimetics, University of Minho, Headquarters of the European Institute of Excellence on Tissue Engineering and Regenerative Medicine, AvePark—Zona Industrial da Gandra, Barco, 4805-017 Guimarães, Portugal; rpirraco@i3bs.uminho.pt (R.P.P.); rgreis@i3bs.uminho.pt (R.L.R.)

**Keywords:** pre-irradiation grafting, P (AAm-co-AN), UCST, thermosensitive, cytotoxicity

## Abstract

In an attempt to find a potential application of cell culture harvesting, a novel method for the preparation of an upper critical solution temperature (UCST) thermosensitive hydrogel was studied. An electron accelerator was used as the electron beam (EB) radiation source, and acrylamide (AAm) was first grafted onto the pre-irradiated polypropylene (PP) sheet. Then, the grafting layer of poly (acrylamide-co-acrylonitrile) (P (AAm-co-AN)) was obtained by the partial dehydration of the acylamino group into the cyano group in the solution mixture of sulfoxide chloride (SOCl_2_) and dimethyl formamide (DMF). The effects of the absorbed dose, AAm concentration, reaction time, and temperature on the degree of grafting were studied, respectively. The effect of the SOCl_2_ concentration on the conversion degree of the cyano group from the acylamino group was studied, followed by the temperature of the UCST. The UCST properties of the grafted samples with P (AAm-co-AN) were studied by quartz crystal microbalance (QCM) and atomic force microscope (AFM), respectively. The cytotoxicities of the hydrogels against cells were verified by CCK-8 studies.

## 1. Introduction

Hydrogels are crosslinked hydrophilic macromolecular networks swollen in water or biological fluid. Generally, thermosensitive hydrogels are responsive to changes in temperature. An important feature of such hydrogels is the presence of a critical solution temperature. When the ambient temperature is above or below this critical solution temperature, the change of the hydrogel’s swelling properties will be obviously different. The change of the solubility is also equivalent to the hydrophilic/hydrophobic transition, such that thermosensitive hydrogels are smart materials that can switch between being hydrophilic and hydrophobic depending on the temperature [1]. When the state of the material is switched between the opposite states of being hydrophilic/hydrophobic, only the temperature of the environment in which the material is located needs to be changed, and then the performance conversion of the material can be achieved by itself, without introducing other processes to transform the material. In recent years, thermosensitive polymers have been widely used in medical devices such as drug release [2,3], tissue engineering [4,5,6], and other biomedical fields [7,8]. From the differences in the thermosensitive characteristics, all those thermosensitive polymers can be defined into two types: polymers with a lower critical solution temperature (the LCST type), which are dissolved in the aqueous solution when the temperature is lower than the LCST, and polymers with an upper critical solution temperature (UCST type), which are dissolved in the aqueous solution when the temperature is higher than the UCST.

Until now, most of the reports of thermosensitive polymers were focused on the LCST type. Among them, poly-*N*-isopropylacrylamide (PNIPAAm) is a typical LCST thermosensitive polymer, the LCST of which is 32 °C. It sharply changes in the state of being a solution/precipitate, and is reversible when the temperature is changed below or above 32 °C [9]. The cell culture in vitro on the surface of PNIPAAm hydrogel is an example of a perfect application of thermosensitive hydrogels. In 1995, Okano [4] first applied PNIPAAm, with an LCST of around 32 °C, which is very close to the physiological temperature of the human body, in cell culture and non-enzymatic harvesting studies. They applied PNIPAAm on the surface of cell culture dishes, and cultured the cell at 37 °C, above the LCST of PNIPAAm; the surface was hydrophobic, which was a benefit to cells for adhering and growing. Then, they decreased the temperature below the LCST, which changed the hydrophobic surface into a hydrophilic surface, and completely peeled off the cell membrane. Compared with the enzymatic hydrolysis method or the cell scraper method, which makes the stemness of stem cells gradually weaken with the increase of the passages, the advantage is that the cells harvested by this method protect and retain the extracellular matrix protein to a greater extent [10,11,12].

A surface with the LCST hydrogel property is suitable for culturing the membrane of a single cell. According to the property of the UCST hydrogel, the cells could adhere to the surface of the hydrogel at a lower temperature, which is hydrophobic below the UCST. The cells could fall off of the surface of the hydrogel at a higher temperature, which is hydrophilic above UCST. It is supposed that the cooperated operation of two kinds of surfaces, which have UCST and LCST hydrogel characteristics, respectively, for harvesting and transferring could be a method for the culturing of multiple cell membranes [13]. However, there are few papers concerning the UCST hydrogels that have been reported, until now [14,15,16]. UCST thermosensitive hydrogels could be divided into two types, according to their functional group of temperature response. One is the zwitterionic type of thermosensitive hydrogels, which is due to the change of the coulombic effect with the temperature, such as polybetaine. The other is non-ionic based UCST hydrogel, and the phase transition could be supposed as the effect of the temperature on the hydrogen bonding between side groups of polymers, such as poly (*N*-acryloylglycinamide) (PNAGA), which was first synthesized by Haas and Schuler [17], and poly (uracilacrylate), which was first reported by Brahme and Smith [18]. These polymers show a UCST in a relevant temperature range (5–60 °C) in aqueous solutions [15]. The UCST behavior of PNAGA in both pure water and electrolyte solution was first reported by Agarwal et al. [19].

According to the research results of Jan Seuring et al. [20], the acrylamide–acrylonitrile system is the most ideal UCST thermosensitive polymer system found so far. This P (AAm-co-AN) hydrogel exhibits an obvious phase transition around the temperature of UCST, and good reversibility of thermosensitive behavior. On the other hand, the easier copolymerization of the two monomers is conducive to the synthesis of copolymers with different composition ratios, which could obtain different targeted UCSTs. Until now, most of the studies on the UCST systems have been concerned with the synthesis methods and the characterization of the solution systems [21,22,23]. There are few reports concerned with the surface properties of UCST hydrogel. Chen et al. reported a smart surface with reversible temperature-dependent wettability properties of the UCST type, which was prepared by coating a UCST P (AAm-co-AN) on the porous anodic aluminum oxide plate (AAO) [24]. Our group reported a thermosensitive hydrogel with UCST behavior, which was prepared by grafting acrylamide (AAm) and acrylic acid (AAc) onto pre-irradiated polypropylene (PP) film [25]. AAm and AAc were grafted onto pre-irradiated PP film using two grafting reactions in aqueous solutions, respectively.

This paper reported a method for the preparation of a UCST thermosensitive hydrogel of poly (acrylamide-co-acrylonitrile) (PP-g-P (AAm-co-AN)), which consisted in grafting AAm onto the pre-irradiated PP sheet, and then partially dehydrating the acrylamino group of the polyacrylamide on the graft layer into the cyano group, in the solution mixture of SOCl_2_-DMF. This kind of UCST hydrogel could have a potential application for cell harvesting in cell culture.

## 2. Experiment

### 2.1. Chemicals and Materials

The acrylamide (Sinopharm Reagent, >99%), acrylonitrile (Sinopharm Reagent, >99%, inhibitor removed), dimethyl sulfoxide (DMSO) (Sinopharm Reagent, >99%), thionyl chloride (Sinopharm Reagent, >99%), *N*,*N*-dimethylformamide (Sinopharm Reagent, >99%) and other solvents were used without further purification. Ultrapure water was obtained from the Sartorius system. A commercial PP sheet with a thickness of 0.3 mm (purchased from market) was used as a substrate for the grafting reaction. The PP sheet was cut into 1 cm × 1 cm pieces and ultrasonically washed twice in acetone/ethanol (1:1) for 1 h each time, and dried in a vacuum oven.

### 2.2. Irradiation

The electron beam (EB) irradiation was carried out under a Dynamitron electrostatic electron accelerator with a dose rate of 200 Gy/s and an energy level of 2.0 MeV [24,25,26,27,28]. The dose range was about 20–200 kGy, and was controlled by the speed of the flat dolly. The irradiated PP sheets were stored in a freezer and kept at −20 °C until the grafting reaction was performed.

### 2.3. Grafting Procedure

The grafting reaction was conducted in a Pyrex ampoule with a cock [24,25,26,27,28]. Water was added first as a solvent, followed by ferrous sulfate (FeSO_4_, 2.5 × 10^−3^ M), sulfuric acid (H_2_SO_4_, 0.2 M), and monomer (AAm). The irradiated sample sheet was immersed in a 10 mL monomer solution mixture in the Pyrex ampoule, and purged with bubbling nitrogen for 20 min. The grafting reaction was carried out by placing the ampoules in a water bath at 60 °C. After grafting, the sample was taken out and washed with ultrapure water at room temperature for 30 min; the water was changed six times. The degree of grafting was determined by the following equation:

(1) $$D_{g}=\frac{W_{g}-W_{0}}{W_{0}}\;\times \;100\%$$

where D_g_ is the grafting degree, *W*_g_ is the weight of PP after it is grafted, and *W*_0_ is the weight of PP before it is grafted.

### 2.4. Dehydration

A solution mixture of SOCl_2_ and DMF was applied for the dehydration of polypropylene grafted with poly (acrylamide) (PP-g-PAAm) into PP-g-P (AAm-co-AN). SOCl_2_ and DMF were mixed in a specific ratio, and the samples of PP-g-PAAm were immersed in the solution to react at 0 °C for 6 h. After the reaction, the samples were taken out and washed using ultrapure water, and were then dried at 50 °C in a vacuum drier for over 12 h. In terms of post-treatment, because SOCl_2_ can react with water and decompose into HCl and SO_2_ when exposed to air, the dehydrating agent will not remain in the sample.

### 2.5. Analytical Techniques

#### 2.5.1. ATR-FTIR Spectroscopy

The ATR-FTIR spectra were recorded using an Avatar 370 Fourier transform infrared spectrometer equipped with an IR light source, and a detector in the range of 550 to 4000 cm^−1^. The measurements were performed using Omnic version 8.2 software (Thermo Electron Corporation, Waltham, MA, USA), and each spectrum was the average of 32 scans. 

#### 2.5.2. Contact Angle Test

The contact angle tests were performed using OCA15EC, a video optical contact angle measuring instrument produced by Dataphysics Instruments GmbH, Germany. A constant-temperature water bath was connected to the measuring table to control the test temperature.

#### 2.5.3. Atomic Force Microscope (AFM)

The changes of the surface roughness of the samples below (at 20 °C) and above (40 °C) the UCST were measured by AFM (Multimode8, Bruker, Germany), respectively. The PP-g-P (AAm-co-AN) sample was soaked in pure water for 1 h, and the AFM measurements were carried out on a heatable sample plate with a drop of water, which supported enough water for the swelling of the sample when it was above the temperature of UCST. The data were analyzed by Nano Scope Analysis 1.5.

#### 2.5.4. Quartz Crystal Microbalance (QCM)

The QCM measurements were carried out using CHI400C (Chenhua Instruments, Shanghai, China). The sample was held on the test plat in a box with filled water, and the temperature was controlled by an external electric silicone heating plate. The determination started at 20 °C, when the vibration went to equilibrium, and was maintained for a certain time. Then the temperature was increased with a step of 5 °C, and the step was adjusted to 1 °C at the sharp changing rate of the vibration, we maintained the equilibrated vibration for a while each time, and this process finished at 40 °C.

#### 2.5.5. Cytotoxicity Test

A Human Kidney Epithelial (293 T) cell line was cultured in Dulbecco’s Modified Eagle Medium (DMEM, Solarbio, Beijing, China) with 10% fetal bovine serum (Thermo Fisher, Waltham, MA, USA) and 1% antibiotic (Solarbio, Beijing, China) in a humidified atmosphere of 5% CO_2_ at 37 °C, with the medium being changed every third day. Cells (at passage 2) were collected by 0.25% trypsin-EDTA (Thermo Fisher, Waltham, MA, USA), and were seeded in a 96 well plate (5 × 10^4^ mL^−1^/well) and cultured in standard conditions until about 70% confluence. The cytotoxicity of Sample 101/23AN (the AAm grafting degree is 101% and the AN concentration of PP-g-P (AAm-co-AN) is 23%) and Sample 98/25AN (the AAm grafting degree is 98%, and the AN concentration of PP-g-P (AAm-co-AN) is 25%) were evaluated with regard to the extraction of the sheet using the cell counting kit (CCK-8, DOJINDO, Kumamoto, Japan). Before the extraction preparation, samples as defined above were sterilized by soaking in 75% ethanol for 30 min, and were washed three times with ultrapure water. Then the samples, 0.5 g each, were soaked in 10 mL medium for 3 days at 37 °C in order to perform the extraction. When the cells reached 70% confluence, the medium was replaced with the extracted medium and cultured for 48 h at 37 °C in order to detect possible cytotoxic effects. The cells cultured with DMEM medium were used as a control. Each sample’s evaluation was performed with five replicates. The absorbance at 450 nm was measured with a microplate reader, and the data were analysed using GraphPad Prism 7; the test method was double-factor variance analysis.

## 3. Results and Discussion

### 3.1. Grafting Reaction

**Figure 1:**
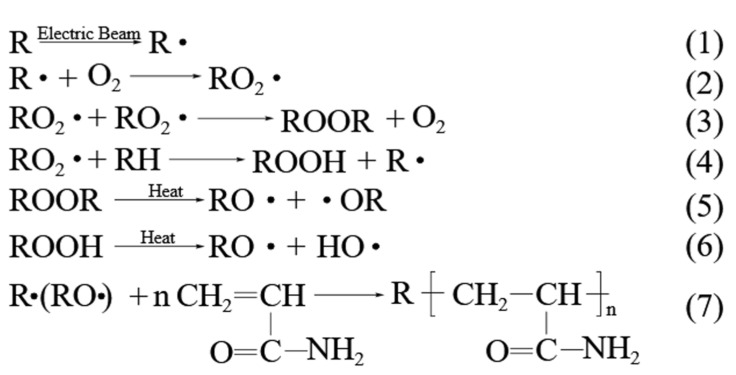
The reaction of the pre-irradiation grafting.

As shown in reaction Formulae (1)–(7) (Figure 1) [26], when the PP sheet is irradiated in nitrogen or air, the free radicals and peroxides capable of initiating the grafting reaction can be formed and retained for a long time, depending on the storage conditions. This means that there is no additional initiator needed for the pre-irradiation grafting. The schematic mechanism of the reaction is as follows:

In these schemes, R represents the polypropylene sheet, and R**·** represents polymeric radicals produced by irradiation. Formulae (1)–(4) are the processed of irradiation and oxidation. Formulae (5) and (6) are the dissociation of the peroxides. Formula (7) is the reaction of AAm grafted onto PP sheets.

### 3.2. Effects of the Absorbed Dose of the Pre-Irradiated PP Sheet, Reaction Temperature, and Monomer Concentration on the Degree of Grafting

**Figure 2:**
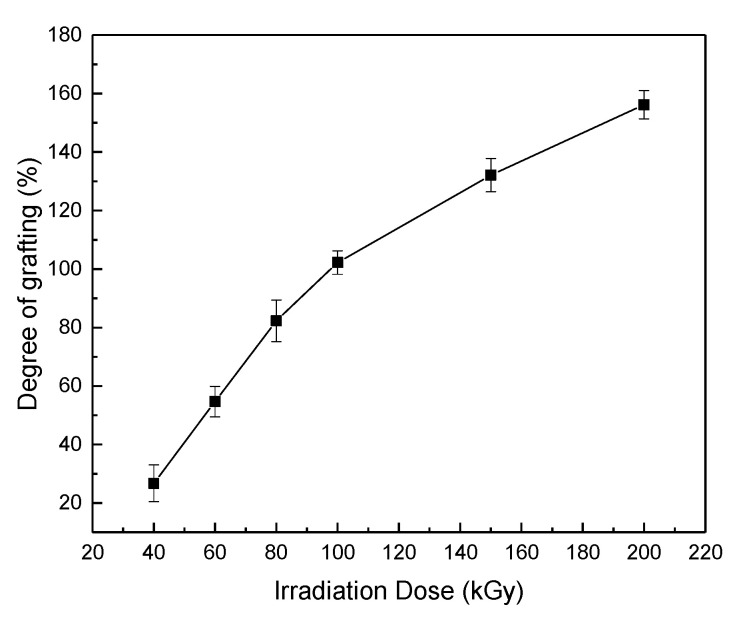
The effect of the absorbed dose of the PP sheets on the degree of grafting (AAm was grafted at 60 °C for 6 h in 30% aqueous solution).

The effect of the absorbed dose of PP sheets on the degree of grafting was studied in an aqueous solution with 0.2 M H_2_SO_4_ and 2.5 × 10^−3^ M Fe^2+^, and the monomer concentration of AAm was 30% *w*/*w*. The reaction was performed in a water bath at 60 °C for 6 h. The results are shown in Figure 2. The degree of grafting increased with the absorbed dose, as usual; the higher the absorbed dose, the more free radicals. When the absorbed dose was up to 200 kGy, the degree of grafting was more than 150%. Sometimes, the grafted samples expanded when the grafting yield was higher than 100%, due to the penetrated grafting in the inner part of the PP sheet. As the result of Figure 2, the grafting reactivity of the PP sheets with the dose of 200 kGy was relatively higher; therefore, the dose of 200 kGy was mainly selected for the grafting reaction in this paper.

**Figure 3:**
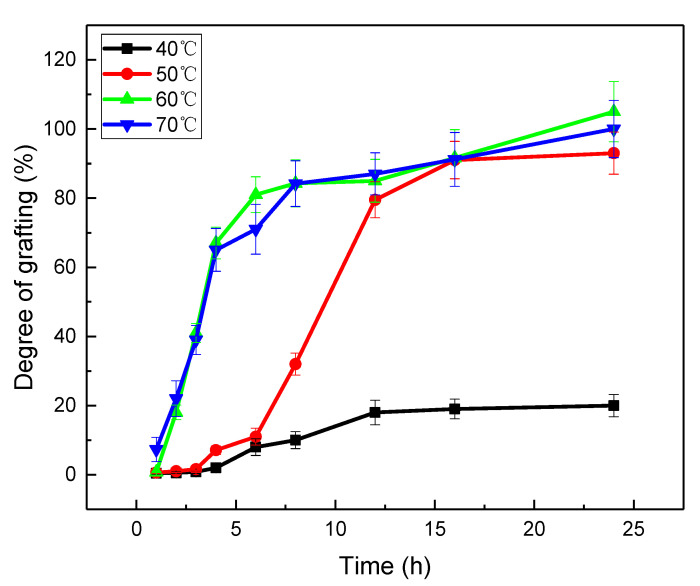
The degree of grafting of AAm versus the reaction time in the comparison of the reaction temperature (AAm was grafted in 25% aqueous solution, and the absorbed dose of the PP sheets was 200 kGy).

Figure 3 is the effect of the reaction time on the degree of grafting in comparison with the reaction temperature. It shows that the degree of grafting of AAm increased with time in each curve. Comparing the reaction temperatures at 40 °C, 50 °C, 60 °C, and 70 °C, it was found that the degree of grafting increased very quickly in the first 4 h, and then levelled off at the reaction temperatures of 60 °C and 70 °C. In the case of 50 °C, the increase of the grafting yield was obviously slower in the beginning, and a sudden rise occurred after 6 h; this then levelled off, and the grafting yield almost arrived in the level of the curves, which were grafted at 60 °C and 70 °C. At 40 °C, the degree of grafting seems obviously lower over the whole 24 h. According to the results in Figure 3, 60 °C is supposed to a more suitable temperature for the reaction than the others.

**Figure 4:**
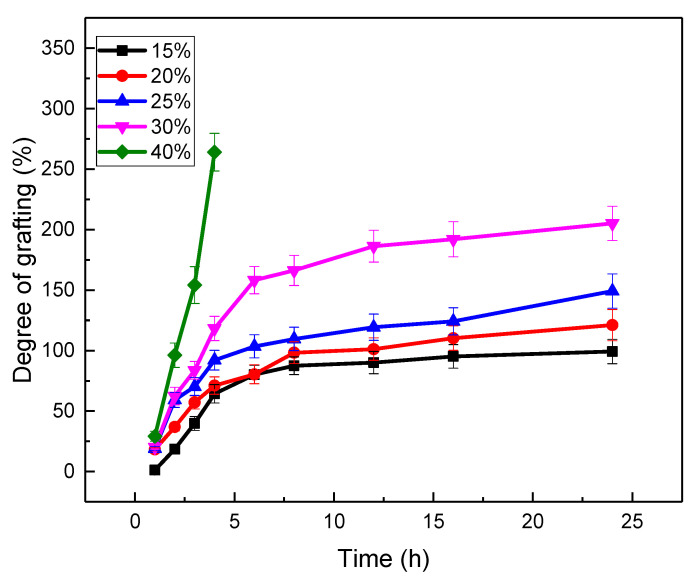
The degree of grafting of AAm versus the reaction time in the comparison of the monomer concentration (AAm was grafted at 60 °C, and the absorbed dose of the PP sheets was 200 kGy).

The degree of grafting of AAm versus the reaction time in the comparison of the monomer concentration was studied at the reaction temperature of 60 °C. The result was that the grafting degree of AAm increased with the increase of the monomer concentration, as shown in Figure 4. With the increase of the monomer concentration, the phenomenon of homopolymerization was becoming more and more serious, particularly when the monomer concentration was above 30% *w/w*. It was found that the residual liquid tended to be gelatinous after the samples underwent a long-term reaction, which decreased the conversion efficiency of the monomer for the grafting. The homopolymers anchored on the surface of the samples were difficult to remove. The optimal degree of grafting was about 100%, which kept the PP sheets flat, and the AN content could be easily controlled in a suitable range during the dehydration. Therefore, a 25% monomer concentration of AAm, which was grafted in water for 6 h at 60 °C, was selected for the reaction.

### 3.3. Dehydration of PP-g-PAAm into PP-g-P (AAm-co-AN)

**Figure 5:**
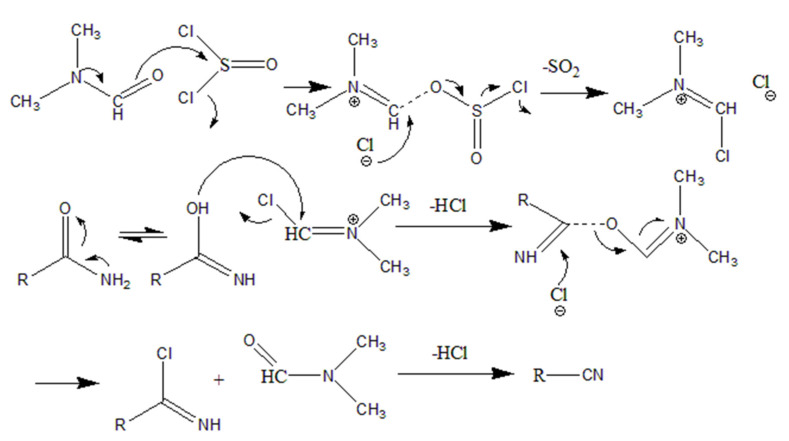
Dehydration reaction of PP-g-PAAm into PP-g-P (AAm-co-AN).

Figure 5 shows the mechanism of the dehydration of the amide groups into cyano groups by a dehydration agent composed of SOCl_2_ and DMF. The process begins with the reaction of SOCl_2_ and DMF: the unshared electron pair of the nitrogen atom in the DMF forms a p-π conjugate with a carbon–oxygen double bond, resulting in the delocalization of the lone pair of electrons on the nitrogen atom. The oxygen atom has a large electronegativity, such that the negative charge is biased towards oxygen. When the oxygen is in an electron-rich state, it will attack SOCl_2_’s electron-deficient sulfur and occupy its empty orbit. Because the chloride anion is more stable than the oxygen anion, and because the degree of sulfur–chlorine bond polarization is large, the sulfur–chlorine bond is broken, and the chlorine anion is removed. As a result of the reaction between SOCl_2_ and DMF, a Vilsmeier intermediate and SO_2_ are formed [27]. The amide group to be dehydrated is reacted with the Vilsmeier intermediate. First, the amide groups become a tautomerism because of the delocalization of the lone pair of electrons. The electron-rich oxygen attacks the electron-deficient carbon in the Vilsmeier intermediate to form a transition state. After removing two molecules of HCl, cyano and DMF are finally formed. According to the analysis of the dehydration mechanism, it can be known that (1) DMF played a catalytic role in this reaction, and (2) there was no stable intermediate product formed during the reaction, which is the theoretical basis for the whole partially dehydrated reaction product as a binary system.

### 3.4. Effect of the SOCl_2_ Ratio in DMF/SOCl_2_ on the Dehydration of AAm

**Figure 6:**
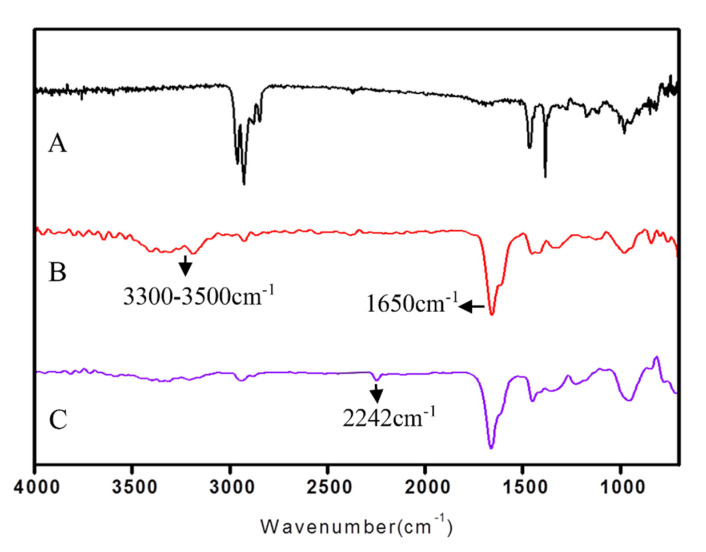
FTIR spectra: (A) blank PP sheet; (B) PP-g-AAm 101%; (C) PP-g-P (P (AAm-co-AN) contains 23% AN). FTIR was applied for the determination of the PAAm:PAN ratio after the dehydration of the AAm-grafted samples.

Figure 6 shows the FTIR spectra of PP, PP-g-PAAm, and PP-g-P (AAm-co-AN), respectively. After grafting, obvious −CONH stretching vibration peaks were generated at 1650 cm^−1^ and 3300–3500 cm^−1^ (B), which proved that AAm had been grafted onto the PP sheet. After the sample was partially dehydrated, a new stretching peak appeared at 2243 cm^−1^ (C). It could be confirmed by comparing the data that it was a −C≡N stretching vibration peak, indicating that the amide group was partially dehydrated to form a cyano group. The target product PP-g-P (AAm-co-AN) was obtained, and the ratio of AAm and AN was estimated according to the peak area of −CONH at 1650 cm^−1^ and −C≡N at 2243 cm^−1^.

**Figure 7:**
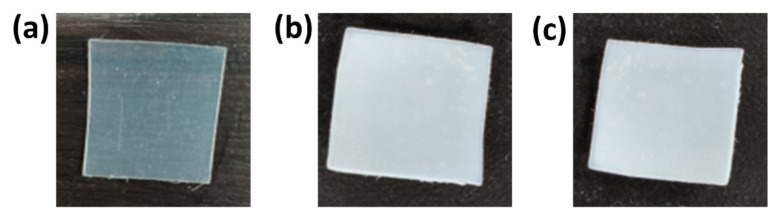
Photographs of the samples before and after the treatment: (**a**) original PP sheet, (**b**) the AAm-grafted PP sheet PP-g-PAAm (101%), and (**c**) PP-g-P (AAm-co-AN) (23% AN).

The photos in Figure 7 are the samples of the original PP sheet, the AAm-grafted PP sheet (PP-g-PAAm) with the degree of grafting of 101%, and the PP-g-P (AAm-co-AN) with the ratio of AN 23%, which is a partial dehydration of the PP-g-PAAm 101% sample, respectively. They show that the original PP sheet was semitransparent, and that it became white and opaque after the graft of AAm. The appearance showed almost no difference between the samples of PP-g-PAAm and PP-g-P (AAm-co-AAm).

**Figure 8:**
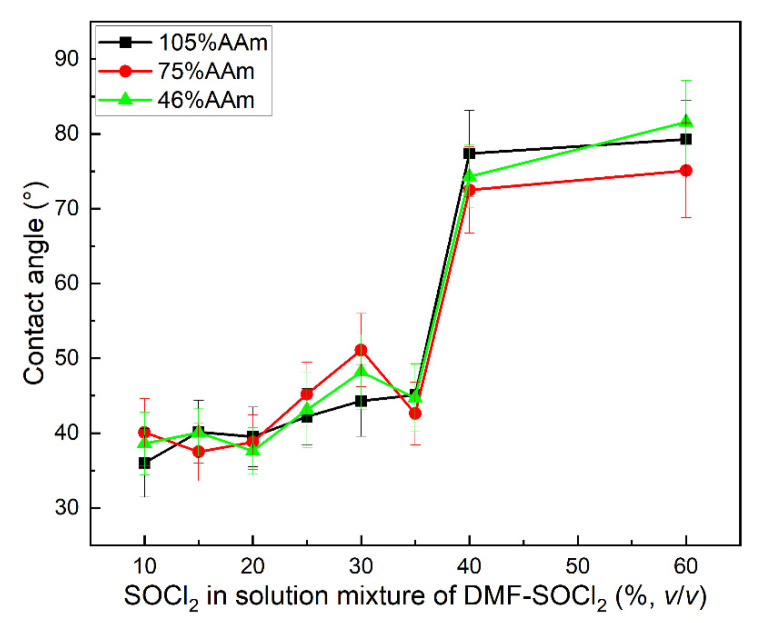
The effect of the SOCl_2_ concentration on the sample’s contact angle.

The water contact angle of the same group of samples was measured at 20 °C, as shown in Figure 8. The sample of PP-g-AAm was hydrophilic, and the water contact angles were around 35°–40°; the water contact angles increased with the dehydration of the acylamino group in the grafted layer due to the hydrophobic of cyano group. When the concentration of SOCl_2_ exceeded 35%, the water contact angle suddenly increased. This means that the copolymer of Poly (AAm-co-AN) was formed, and that the hydrophilic surface of PP-g-AAm was changed into hydrophobic PP-g-P (AAm-co-AN).

## 4. UCST Test

**Figure 9:**
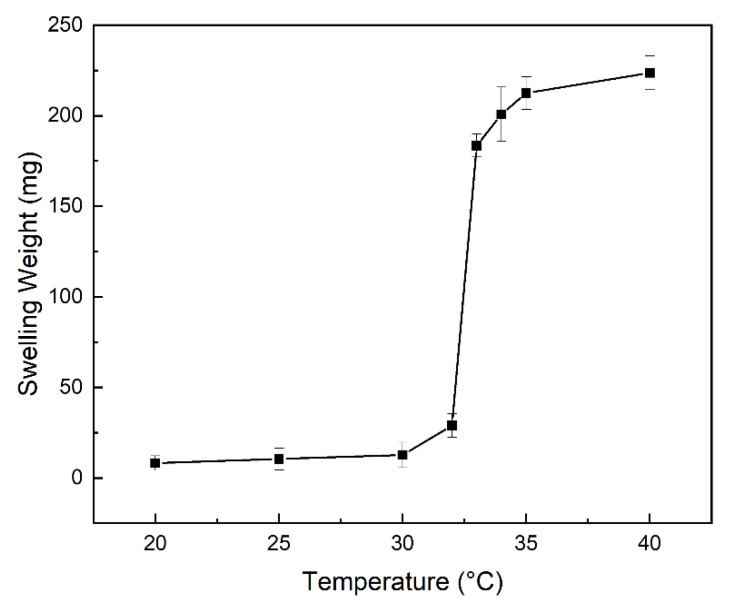
The relationship between the temperature and swelling weight. The AAm grafting degree is 101% of PP-g-PAAm, and the AN concentration of PP-g-P (AAm-co-AN) is 23%.

The relationship between the swelling weight of the PP-g-P (AAm-co-AN) sample and the temperature was ascertained by quartz crystal microbalance, as shown in Figure 9. The swelling weight showed almost no change before 30 °C; however, it sharply increased when the temperature was above 30 °C, and then levelled off around 33 °C. This result obviously indicated that the PP-g-P (AAm-co-AN) sample possesses the property of UCST thermosensitive hydrogel, and that the UCST temperature for the sample of PP-g-P (AAm-co-AN) with the grafting yield of PAAm 101% and PAN content of 23% was 32.5 °C. In the case of PP-g-PAAm, we did not find the change of the swelling weight with the temperature in the same test. This means that the PP-g-PAAm sample had no UCST property before the dehydration. PP-g-P (AAm-co-AN) samples with a grafting degree of AAm 101% and different AN contents were determined by the same method, respectively, and the result of the UCST temperature is shown in Table 1.

It seems that the UCST temperature increased with the contents of AN in the graft layer. However, when the content of AN was above 31.93%, it was no longer thermosensitive. These results are similar to the block copolymer of P (AAm-co-AN), as reported by Hu Zhang et al. [25].

**Figure 10:**
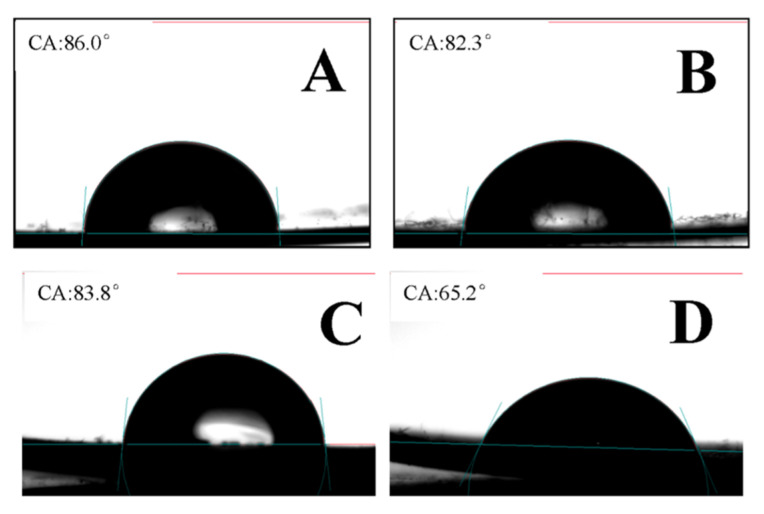
Contact angle test: (**A**) the blank PP sheet at 20 °C, (**B**) the blank PP sheet at 40 °C, (**C**) the sample at 20 °C, and (**D**) the sample at 40 °C (the AAm grafting degree is 101%, and the AN concentration of PP-g-P (AAm-co-AN) is 23%).

The water contact angle test is one of the most intuitive and convenient methods for the determination of the surface property. Figure 10 shows the photos of the water contact angle of the PP-g-P (AAm-co-AN) sample with 77% amide and 23% AN, which was obtained via the dehydration of the grafted sample with 101% AAm. When the temperature of the sample was raised from 20 °C to 40 °C, the contact angle decreased from 83.8° to 65.2°, which meant that the surface of the sample changed relatively from being hydrophobic to hydrophilic. It could be illustrated that the non-ionic-based UCST polymers relied on hydrogen bonding between polymer side groups for the phase transition. When the temperature increased up to the UCST, the hydrogen bonds between the polymer side groups were broken in an endothermic process, and were replaced by hydrogen bonds with water molecules in an exothermic process [23].

**Figure 11:**
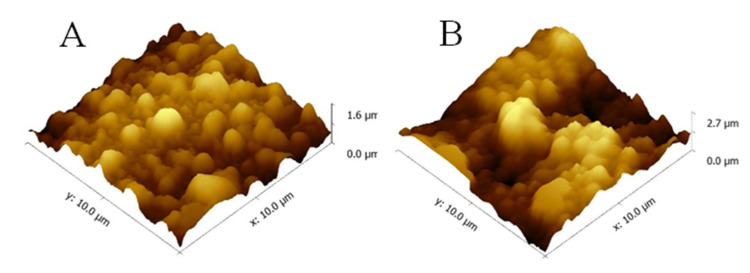
AFM morphologies of the PP-g-P (AAm-co-AN) sample in a plate with water at different temperatures: (**A**) at 20 °C, and (**B**) at 40 °C. The AAm grafting degree is 101%, and the AN concentration of PP-g-P (AAm-co-AN) is 23%.

The UCST behavior of the PP-g-P (AAm-co-AN) sample with the grafting degree of AAm 101% and AN contents of 23% was studied using an AFM at temperatures of 20 °C and 40 °C, respectively, as shown in Figure 11. This clearly showed that the surface of the sample was much rougher at 40 °C than it was at 20 °C. This result could be explained by the fact that the swelling ratio of the sample was much higher at 40 °C, which was above its UCST.

## 5. Cytotoxicity Studies

**Figure 12:**
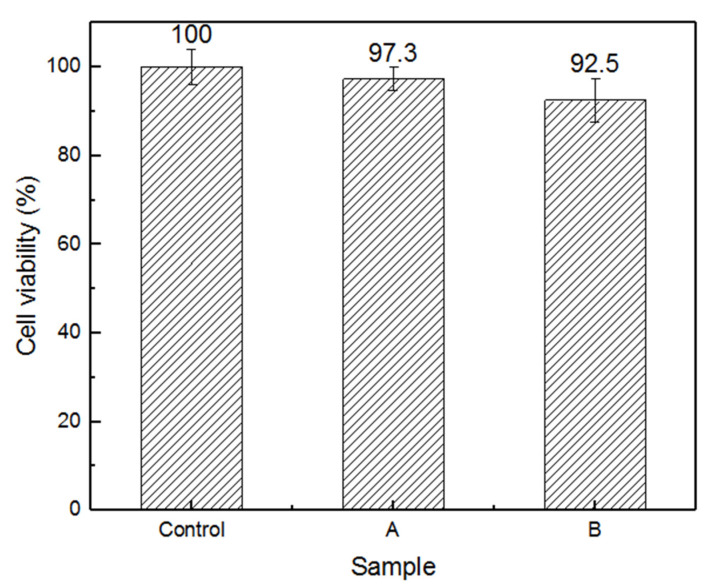
In vitro cytotoxicity of the PP-g-P (AAm-co-AN) after incubation with cells for 48 h at 37 °C. (A) The AAm grafting degree is 101%, and the AN concentration of PP-g-P (AAm-co-AN) is 23%. (B) The AAm grafting degree is 98%, and the AN concentration of PP-g-P (AAm-co-AN) is 25%. The control was the cells cultured with DMEM medium. The ANOVA test showed that there was no significant difference between the control, sample A and sample B.

In order to assess the suitability of the samples as biomaterials for cell harvest and transfer, in this study, the cytotoxicity of PP-g-P (AAm-co-AN) was tested using a CCK-8 assay, which is based on the ability of succinate dehydrogenase in the mitochondria of viable cells to reduce the exogenous CCK-8 to water-insoluble orange crystal formazan. The color depth is directly proportional to the cell proliferation, and is inversely proportional to the cytotoxicity. Figure 12 is the arithmetic average value of the data obtained; it shows that the survival rates of the two groups of samples after 48 h of culturing were not less than 90% compared with the control group, suggesting that the surface modification doesn’t induce cytotoxicity. These results preliminarily confirm the possibility of the use of PP-g-P (AAm-co-AN) as a safe carrier to harvest and transfer cells.

## 6. Conclusions

A grafted hydrogel layer of P (AAm-co-AN) with UCST properties was successfully obtained by the pre-irradiation method. An EB accelerator was applied as a radiation source, and 200 kGy was selected as the suitable dose. AAm was first grafted onto the PP sheet, and then the PP-g-P (AAm-co-AN) with UCST hydrogel properties was obtained by the partial dehydration of the acylamino group into the cyano group in the solution mixture of DMF-SOCl_2_. The results showed that the samples with different UCST temperatures could be obtained by adjusting the percentage of AAm dehydration, which means the AN content. The optimum hydrogel surface, which appeared sharper with the changing of the swelling behavior at around 32 °C, was the sample with a grafting yield of AAm of around 101% and a dehydration of 23%. Accordingly, the optimum grafting condition for 101% PP-g-PAAm was found in the 25% AAm aqueous solution at 60 °C for 6 h, and was dehydrated in the solution mixture of DMF:SOCl_2_ 60:40 at 0 °C for 6 h. The CCK-8 tests revealed that there was no noticeable cytotoxicity of PP-g-P (AAm-co-AN) against the 293 T cell line. In summary, such a kind of UCST smart hydrogels could have excellent prospects in biomedical science, cell harvesting, and other applied fields.

## Figures and Tables

**Table 1:** UCST of samples with different AN contents.

AN (%)	UCST (°C)
16.22	25.5
20.14	30.5
22.86	32.5
26.53	40.5
30.66	49.5
31.93	54.5

## Data Availability

No data availability statement.
